# BCL2 in breast cancer: a favourable prognostic marker across molecular subtypes and independent of adjuvant therapy received

**DOI:** 10.1038/sj.bjc.6605736

**Published:** 2010-07-27

**Authors:** S-J Dawson, N Makretsov, F M Blows, K E Driver, E Provenzano, J Le Quesne, L Baglietto, G Severi, G G Giles, C A McLean, G Callagy, A R Green, I Ellis, K Gelmon, G Turashvili, S Leung, S Aparicio, D Huntsman, C Caldas, P Pharoah

**Affiliations:** 1 Department of Oncology, University of Cambridge, Cambridge CB1 9RN, UK; 2 Cancer Research UK Cambridge Research Institute, Li Ka Shing Centre, Robinson Way, Cambridge CB2 ORE, UK; 3 Strangeways Research Laboratories, University of Cambridge, Cambridge CB1 9RN, UK; 4 Cambridge Breast Unit, Addenbrooke's Hospital, Cambridge University Hospital NHS Foundation Trust and NIHR Cambridge Biomedical Research Centre, Cambridge CB2 2QQ, UK; 5 Cancer Epidemiology Centre, The Cancer Council Victoria, Carlton, Victoria 3053, Australia; 6 Centre for Molecular, Environmental, Genetic, and Analytic Epidemiology, University of Melbourne, Parkville, Victoria 3010, Australia; 7 Department of Anatomical Pathology, The Alfred Hospital, Melbourne, Victoria 3181, Australia; 8 Department of Pathology, NUI, Galway, Ireland; 9 Department of Histopathology, Nottingham City Hospital, Nottingham NG5 1PB, UK; 10 Genetic Pathology Evaluation Centre of the Department of Pathology and Prostate Research Centre, Vancouver General Hospital, British Columbia Cancer Agency and University of British Columbia, Vancouver, British Columbia, Canada V6H 3Z6

**Keywords:** BCL2, breast cancer, prognosis

## Abstract

**Background:**

Breast cancer is heterogeneous and the existing prognostic classifiers are limited in accuracy, leading to unnecessary treatment of numerous women. B-cell lymphoma 2 (BCL2), an antiapoptotic protein, has been proposed as a prognostic marker, but this effect is considered to relate to oestrogen receptor (ER) status. This study aimed to test the clinical validity of BCL2 as an independent prognostic marker.

**Methods:**

Five studies of 11 212 women with early-stage breast cancer were analysed. Individual patient data included tumour size, grade, lymph node status, endocrine therapy, chemotherapy and mortality. BCL2, ER, progesterone receptor (PR) and human epidermal growth factor receptor 2 (HER2) levels were determined in all tumours. A Cox model incorporating the time-dependent effects of each variable was used to explore the prognostic significance of BCL2.

**Results:**

In univariate analysis, ER, PR and BCL2 positivity was associated with improved survival and HER2 positivity with inferior survival. For ER and PR this effect was time dependent, whereas for BCL2 and HER2 the effect persisted over time. In multivariate analysis, BCL2 positivity retained independent prognostic significance (hazard ratio (HR) 0.76, 95% confidence interval (CI) 0.66–0.88, *P*<0.001). BCL2 was a powerful prognostic marker in ER− (HR 0.63, 95% CI 0.54–0.74, *P*<0.001) and ER+ disease (HR 0.56, 95% CI 0.48–0.65, *P*<0.001), and in HER2− (HR 0.55, 95% CI 0.49–0.61, *P*<0.001) and HER2+ disease (HR 0.70, 95% CI 0.57–0.85, *P*<0.001), irrespective of the type of adjuvant therapy received. Addition of BCL2 to the Adjuvant! Online prognostic model, for a subset of cases with a 10-year follow-up, improved the survival prediction (*P*=0.0039).

**Conclusions:**

BCL2 is an independent indicator of favourable prognosis for all types of early-stage breast cancer. This study establishes the rationale for introduction of BCL2 immunohistochemistry to improve prognostic stratification. Further work is now needed to ascertain the exact way to apply BCL2 testing for risk stratification and to standardise BCL2 immunohistochemistry for this application.

The management of breast cancer continues to be challenging because of the heterogeneity of the disease. In early-stage breast cancer, a limited number of clinical and pathological factors are currently used to guide prognosis. These factors include age, tumour size, histological grade, lymphovascular invasion and oestrogen receptor (ER) status, and have been incorporated into algorithms such as the Nottingham Prognostic Index or Adjuvant! Online (AOL) to estimate the individual risk (Haybittle *et al*, 1982; Ravdin *et al*, 2001; Blamey *et al*, 2007). More recently, amplification and/or overexpression of the human epidermal growth factor receptor 2 (HER2), a therapeutic target, has been associated with worse prognosis, although its clinical utility as a prognostic marker remains uncertain (Paik *et al*, 1990; Yamauchi *et al*, 2001; Chia *et al*, 2008). The variation in clinical outcome despite similar prognostic scores seriously compromises our ability to advise women in making informed decisions about adjuvant treatment.

Over several decades, substantial effort has been invested in the identification and validation of additional prognostic markers to improve risk stratification for breast cancer. Despite this, most candidate-based prognostic markers, with the exception of urokinase plasminogen activator and plasminogen activator inhibitor 1, have not succeeded in making the transition from the laboratory to clinical practice, as evidenced by the 2007 American Society of Clinical Oncology update on recommendations for the use of tumour markers for breast cancer (Harris *et al*, 2007). As the evaluation of candidate prognostic markers is often limited by inadequate study design and analyses, formal recommendations for reporting tumour marker prognostic studies (REMARK) have now been agreed upon (McShane *et al*, 2005).

In recent years, microarray-based technology has resulted in the identification of breast cancer molecular subtypes (luminal, HER2-like, basal/triple negative) and gene-expression prognostic signatures (Van't Veer *et al*, 2002; Sorlie *et al*, 2003; Paik *et al*, 2006). These prognostic expression signatures hold great promise, but there are concerns regarding their significance independent of ER status and their time dependency (Cardoso *et al*, 2008; Sparano and Paik, 2008). The process of validating the clinical utility of two such expression signatures, Oncotype DX and Mammaprint, is ongoing through the TAILORx and MINDACT trials, respectively (Cardoso *et al*, 2008; Sparano and Paik, 2008). The results from these large randomised prospective trials will be unavailable for many years, and the technology is cumbersome, which may limit its suitability for routine use in clinical practice. In contrast, immunohistochemistry is well established in routine diagnostic pathology laboratories.

Analysis of protein expression using immunohistochemistry has identified molecular subtypes that are similar to those derived from gene expression arrays (Callagy *et al*, 2003; Abd El-Rehim *et al*, 2005). Our group previously assessed the prognostic value of combining protein markers used to define these molecular subtypes. Indeed, we showed that only BCL2 added prognostic information independent of the Nottingham Prognostic Index and validated this result (Callagy *et al*, 2006). Expression of BCL2, an antiapoptotic protein, is associated with low-grade, slowly proliferating, ER+ breast tumours (Silvestrini *et al*, 1994; Lipponen *et al*, 1995). Previous studies had identified that expression of BCL2 was associated with improved survival from breast cancer, but this was attributed to its correlation with ER status (Berardo *et al*, 1998; Charpin *et al*, 1998; Callagy *et al*, 2006, 2008; Neri *et al*, 2006). The aim of the current study was to prospectively test the clinical validity of BCL2 as a prognostic marker independent of ER, HER2 and adjuvant therapy received, in addition to tumour size, grade and nodal status.

## Methods

### Study population, tumour samples, immunostaining and scoring

We have followed the REMARK guidelines for conducting this tumour marker study (Supplementary Table 1) (McShane *et al*, 2005). A total of 11 212 early-stage breast cancer cases were participants in five large studies (all approved by the relevant institutional review boards or ethics committees): the Study of Epidemiology and Risk Factors in Cancer Heredity (SEARCH; *n*=3420), the Nottingham Breast Cancer Series (NBCS; *n*=1926), the University of British Columbia Breast Cancer Series (UBCBCS; *n*=976), the British Columbia Cancer Agency Case Series (BCCA; *n*=4040) and the Melbourne Collaborative Cohort Study (MCCS; *n*=850). The criteria for inclusion were the availability of tumour tissue, pathological data (tumour size, tumour grade, lymph node status) and individual clinical outcome data (vital status at last follow-up and date of death). Early-stage breast cancer was defined as stage I to stage III as per the American Joint Committee on Cancer (AJCC) (2002). Details of the studies have been published previously and a summary of the individual studies is given in Supplementary Table 2 (Ragaz *et al*, 1997, 2005; Giles and English, 2002; Abd El-Rehim *et al*, 2004; Lesueur *et al*, 2005; Callagy *et al*, 2006; Chia *et al*, 2008). Tissue microarrays were constructed for the SEARCH, NBCS, UBCBCS and BCCA studies, as published previously (Kononen *et al*, 1998; Cheang *et al*, 2006). For the MCCS study, whole-tissue sections rather than tissue microarrays were used. Using these samples, we obtained ER, progesterone receptor (PR), HER2 and BCL2 data from 8310 cases (three studies) and pooled it with our previously published data (two studies) for the same markers from a further 2902 women. Details of the immunostaining are given in Supplementary Methods, along with the scoring methods used for ER, PR, HER2 and BCL2 in the different series.

### Statistical analysis

Statistical analysis was conducted in concordance with recently published guidelines for the development of prognostic models in breast cancer (Altman, 2009). Cox regression analysis stratified by study was performed to determine the effect of each prognostic factor and marker on survival after diagnosis. In univariate analysis, inspection of standard log–log plots showed that the Cox proportional hazards assumption was clearly violated for all variables, indicating that the hazard ratio (HR) varied with time (data not shown). We therefore fitted Cox models in which the HR was allowed to vary as a function of time (TV) by extending the basic Cox model to include a time-dependent coefficient in the linear predictor *X*. The log HR (HR_TV_) for any variable (*X*) at time (*t*) is then as follows: log (HR_TV_)=(*B*_1_+*B*_2_ × log (*t*)) *X*. The Cox model allowing for time dependence generates two parameter estimates for each variable (*X*): *B*_1_ (log HR) and *B*_2_ (log time effect (*T*)). If there is no time dependence, *B*_2_ equals zero. If the HR increases with time, *B*_2_ will be positive, and if the HR decreases with time, *B*_2_ will be negative. A comparison of the fit of the time-dependent models compared with the basic Cox models was performed using a likelihood ratio test, and the fit of the time-dependent model was significantly better than the basic model for all variables. On the basis of model likelihoods, the best-fitting model for tumour size, grade and nodal status, PR, HER2 and BCL2 was one with the HR varying as a function of log(time), whereas for ER, the best-fitting model varied as a linear function of time, although this was not substantially better than the model with the HR varying as a function of log(time). Therefore, log(time) was used in subsequent analyses. The value of adding BCL2 to the prognostic model was tested using both receiver operating characteristic (ROC) and relative utility curve analyses (Pepe *et al*, 2004; Baker, 2009). Further details of the statistical analysis are given in Supplementary Methods.

## Results

### Case and tumour characteristics of 11 212 women with early breast cancer

**Figure 1:**
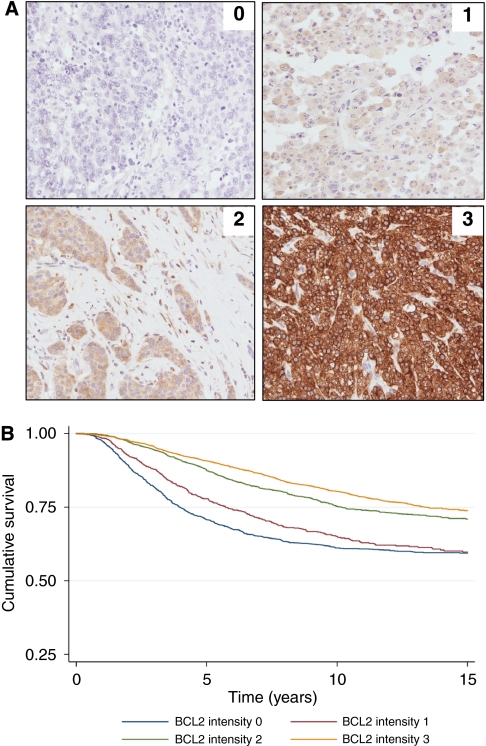
Prognostic significance of BCL2 according to the level of BCL2 expression. (**A**) Immunohistochemical evaluation of BCL2 staining intensity. Immunohistochemical analysis of BCL2 from the SEARCH series (BCL2 antibody, Dako clone 124, 1 : 200). (**B**) Kaplan–Meier curve of cumulative survival according to BCL2 staining intensity. 0=no BCL2 staining: *n*=1646, HR=1.00; 1=weak BCL2 staining intensity: *n*=1100, HR=0.76, 95% CI 0.66–0.88, *P*<0.001; 2=moderate BCL2 staining intensity: *n*=1921, HR=0.55, 95% CI 0.49–0.63, *P*<0.001; 3=strong BCL2 staining intensity: *n*=3022, HR=0.45, 95% CI 0.40–0.51, *P*<0.001.

Baseline clinical and pathology data from the 11 212 women are summarised in Table 1. Differences in recruitment criteria between each of the five cohorts resulted in some variability in the subject's characteristics (Table 1). The UBCBCS series comprised women, all of whom received adjuvant chemotherapy in the setting of clinical trials, whereas the other four studies were population-based cohorts of women with early-stage breast cancer, for whom adjuvant systemic treatment decisions were taken according to the standard clinical guidelines at the time. For this reason, a higher proportion of women in the UBCBCS series had ER−, node-positive tumours, with higher annual mortality rates. The mean follow-up of the study population was 8.4 years. In total, 71% were ER+, 56% were PR+ and 14% were HER2+. Positive BCL2 expression was identified in 73% of cases (86% of these were ER+ and 8% were HER2+). The majority of BCL2+ cases demonstrated moderate-to-strong BCL2 staining intensity (Figure 1) and a high percentage of cells stained positively (Supplementary Figure 1).

### Increasing expression levels of BCL2 predict better survival in early breast cancer

The impact of differential BCL2 expression was explored by comparing the HR for women with tumours showing varying levels of BCL2 staining intensity using a simple univariate analysis (Figure 1A). A direct relationship between the intensity of BCL2 staining and survival was identified (Figure 1B). Women whose tumours demonstrated the most intense BCL2 staining had the best survival.

### BCL2 is a time-independent good prognostic marker in early breast cancer

Univariate analysis using the Cox model allowing for time dependence indicated that the HRs for all variables showed time-dependent effects (Table 2). In an initial multivariate model, which included ER, PR, HER2 and BCL2 (Table 2; model 1), a time-dependent relationship was identified for ER and PR, but not for BCL2 or HER2. In particular, for ER status, a significant increase in HR was noted over time. ER positivity was associated with a favourable prognostic effect for the first 4 years of follow-up (i.e., HR<1.0), but thereafter, ER positivity was associated with an adverse outcome and increased risk of death (i.e., HR>1.0) (Supplementary Figure 2A). In contrast, BCL2 positivity continued to be associated with a favourable prognostic effect throughout the follow-up period.

The time-dependent effects on HRs can be explained by the fact that the mortality rate in ER− disease peaks in the initial 2 years after diagnosis, followed by a steady decline over time (Supplementary Figure 2B). In contrast, although the peak mortality rate for those with ER+ disease is considerably lower in the first few years after diagnosis, there is little change in this mortality rate over time. Eventually, with long-term follow-up, the mortality rate for ER− disease falls below the mortality rate for ER+ disease (Supplementary Figure 2B). A similar trend is noted in the mortality rate for those with BCL2− *vs* BCL2+ disease, but the variation is not as marked and the mortality rate for BCL2+ disease continues to be lower than that for BCL2− disease over time (Supplementary Figure 2B). Therefore, unlike ER positivity, BCL2 positivity retains its favourable prognostic effect with long-term follow-up.

### BCL2 is a prognostic marker independent of clinical–pathological characteristics, molecular subtypes and adjuvant therapy

In a final multivariate model containing tumour size, grade, nodal status, ER, PR, HER2 and BCL2 status, a time-dependent relationship was confirmed for all variables except tumour size, HER2 and BCL2. The most pertinent model was therefore one that included the main effect for tumour size, grade, nodal status, ER, PR, HER2 and BCL2, in addition to time-dependent effects for all variables except tumour size, HER2 and BCL2 (Table 2; model 2). In this model, BCL2 remained an independent predictor of survival (BCL2+ *vs* BCL2− HR 0.76, 95% confidence interval (CI) 0.66–0.88, *P*=0.0002).

**Figure 2:**
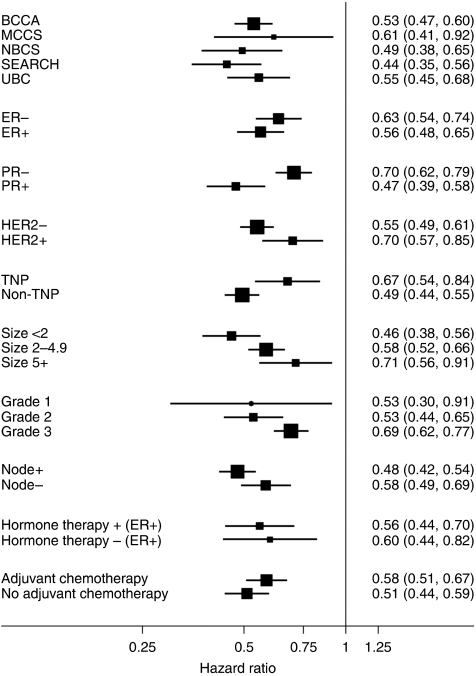
Prognostic significance of BCL2 according to study, tumour characteristics and type of adjuvant therapy. Plot showing the hazard ratio and 95% confidence intervals for BCL2 positivity according to the individual study, tumour characteristics (ER, HER2, TNP (triple-negative phenotype), tumour grade, lymph node status, tumour size) and the type of adjuvant therapy received.

BCL2 was found to be prognostic in each of the five studies analysed (Figure 2). Furthermore, given that the prognostic impact of BCL2 had initially been determined in the UBCBCS series (previously published data) (Callagy *et al*, 2006), the current model was assessed in the remaining four studies to independently validate the original findings (BCL2+ *vs* BCL2− HR 0.79, 95% CI 0.68–0.93, *P*=0.003) (Supplementary Table 3).

**Figure 3:**
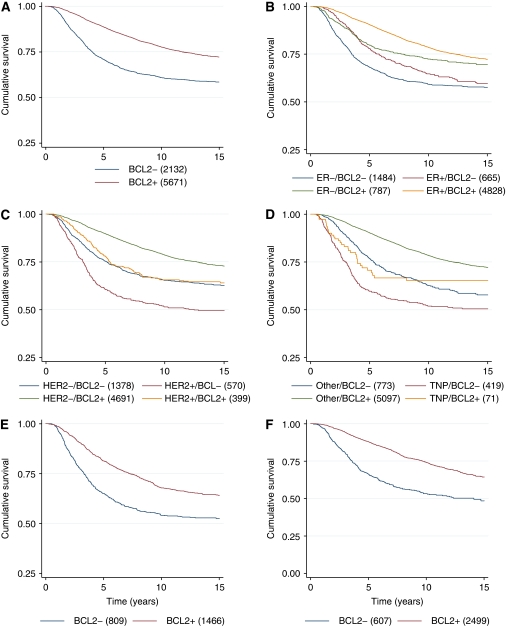
Prognostic significance of BCL2 according to hormonal status, HER2 status and adjuvant therapy. Kaplan–Meier curves of cumulative survival according to (**A**) BCL2 status: (i) BCL2−, HR=1.00; (ii) BCL2+, HR=0.76, 95% CI 0.66–0.88, *P*<0.001. (**B**) Oestrogen receptor and BCL2 status: (i) ER−/BCL2−, HR=1.00; (ii) ER+/BCL2−, HR=0.81, 95% CI 0.69–0.96, *P*=0.012; (iii) ER−/BCL2+, HR=0.62, 95% CI 0.53–0.71, *P*<0.001; (iv) ER+/BCL2+, HR=0.46, 95% CI 0.42–0.51, *P*<0.001. (**C**) Human epidermal growth factor receptor 2 and BCL2 status: (i) HER2−/BCL2−, HR=1.00; (ii) HER2+/BCL2−, HR=1.36, 95% CI 1.17–1.59, *P*<0.001; (iii) HER2−/BCL2+, HR=0.55, 95% CI 0.49–0.61, *P*<0.001; (iv) HER2+/BCL2+ *n*=399, HR=0.94, 95% CI 0.78–1.13, *P*=0.52. (**D**) Triple-negative phenotype (TNP) and BCL2 status: (i) non-TNP/BCL2−, HR=1.00; (ii) TNP/BCL2−, HR=1.43, 95% CI 1.18–1.74, *P*<0.001; (iii) non-TNP/BCL2+, HR=0.56, 95% CI 0.49–0.65, *P*<0.001; (iv) TNP/BCL2+, HR=1.19, 95% CI 0.83–1.73, *P*=0.34. (**E**) Adjuvant chemotherapy and BCL2 status: (i) adjuvant chemotherapy/BCL2−, HR=1.00; (ii) adjuvant chemotherapy/BCL2+, HR=0.76, 95% CI 0.71–0.82, *P*<0.001. (**F**) Adjuvant endocrine therapy and BCL2 status: (i) adjuvant endocrine therapy/BCL2−, HR=1.00; (ii) adjuvant endocrine therapy/BCL2+, HR=0.69, 95% CI 0.64–0.75, *P*<0.001.

The prognostic impact of BCL2 positivity remained regardless of other tumour characteristics, including tumour size, grade and lymph node status (Figure 2). BCL2 was a prognostic factor in women with both ER− (HR 0.63, 95% CI 0.54–0.74, *P*<0.001) and ER+ disease (HR 0.56, 95% CI 0.48–0.65, *P*<0.001) (Figures 2 and 3B). It is of importance that women with ER+/BCL2− disease were found to have a worse prognosis than those with ER−/BCL2+ disease (HR 1.35, 95% CI 1.10–1.65, *P*=0.004). BCL2 was also a strong prognostic marker in women with both HER2− (HR 0.55, 95% CI 0.49–0.61, *P*<0.001) and HER2+ disease (HR 0.70, 95% CI 0.57–0.85, *P*<0.001) (Figures 2 and 3C) and women with triple-negative disease (i.e., ER−, PR− and HER2−) (HR 0.67, 95% CI 0.54–0.84, *P*<0.001) (Figures 2 and 3D).

Finally, the prognostic impact of BCL2 remained irrespective of whether women had received adjuvant chemotherapy (HR 0.58, 95% CI 0.51–0.67, *P*<0.001) (Figures 2 and 3E) or adjuvant endocrine therapy (HR 0.56, 95% CI 0.44–0.70, *P*<0.001) (Figures 2 and 3F).

### BCL2 inclusion as a prognostic marker improves survival prediction

To further assess the final prognostic model (model 2), we used grade, stage, nodal status, ER, PR and BCL2, in addition to time-dependent effects for all variables except nodal status and BCL2, to predict the expected number of deaths across all five studies. The area under the ROC curve was assessed to compare the performance of the predictive model with and without the inclusion of BCL2 (Pepe *et al*, 2004). An improvement in the prediction of the model was observed with the addition of BCL2 (area under ROC 0.6844 with BCL2 *vs* 0.6774 without BCL2, *P*=0.012).

**Figure 4:**
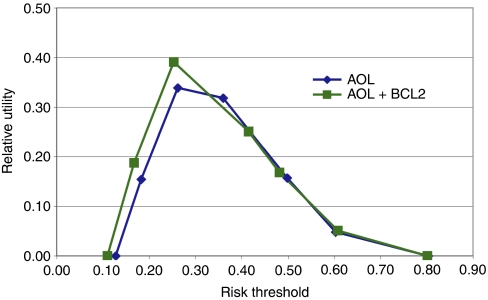
BCL2 as a prognostic marker for risk prediction. Relative utility curves comparing the performance of the risk prediction algorithm used by Adjuvant! Online (AOL) with the performance of AOL in combination with BCL2.

Furthermore, the addition of BCL2 to the risk prediction provided by AOL was assessed in the BCCA series, for which we had 10-year follow-up data. The area under the ROC curve was significantly improved when BCL2 was added to the AOL risk prediction algorithm compared with the use of AOL alone (area under ROC 0.7224 for BCL2 and AOL *vs* 0.7137 for AOL, *P*=0.0039). The improved performance of the AOL risk prediction algorithm with the addition of BCL2 was also confirmed using a relative utility curve (Figure 4) (Baker, 2009). The relative utility is the fraction of the expected utility of perfect prediction achieved at the optimal cutoff point for a risk prediction model. Of particular note is that the relative utility of AOL and BCL2 was superior to the performance of AOL alone at lower risk thresholds at which clinical decision making regarding the use of adjuvant chemotherapy is more difficult.

## Discussion

A great challenge in breast cancer management is to identify patients who will not benefit from systemic adjuvant chemotherapy. Current approaches are hindered by a dearth of clinically useful biomarkers, with the exception of ER and possibly HER2/neu. Although promising, the readiness of gene expression signatures to further stratify patients awaits the results of prospective studies. In contrast, immunohistochemical analysis of BCL2 protein expression is a simple, well-validated, inexpensive and widely available test (used routinely in diagnostic pathology of low-grade lymphoproliferative disorders). The prospective analysis in over 11 000 women with early-stage breast cancer reported here demonstrates for the first time the robust prognostic significance of BCL2 protein expression independent of ER, as well as all the other traditional prognostic markers used in clinical practice (see Figure 2) (Hayes *et al*, 1996).

Significant temporal variation exists in the relative contribution of individual prognostic markers during prolonged follow-up, further limiting their clinical value. Our analysis confirms the previously described time dependence of hormone receptors (ER and PR) as prognostic markers in breast cancer (Hilsenbeck *et al*, 1998; Anderson *et al*, 2006). This time dependence has also been noted in gene expression signatures that appear to be better predictors of relapse in the first 5 years (Desmedt *et al*, 2007; Cardoso *et al*, 2008). In contrast, our study reveals that the prognostic effect of BCL2 protein expression is time independent and BCL2 continues to be associated with favourable outcome over time, increasing its potential clinical value given the frequent occurrence of late relapses (particularly in ER+ breast cancer).

BCL2 belongs to a group of related proteins that are key regulators of apoptosis or programmed cell death (Cory *et al*, 2003). The tumourigenic potential of inappropriate BCL2 protein expression was first described as a result of the chromosomal translocation (*t*(14,18)) seen in subsets of non-Hodgkin's lymphoma, in which it is associated with adverse outcome (Tsujimoto *et al*, 1984). Since this discovery, overexpression of BCL2 protein has been identified in a variety of solid organ malignancies, including breast cancer. In contrast to non-Hodgkin's lymphoma, BCL2 protein expression in breast cancer is associated with an indolent phenotype of low-grade, slowly proliferating, ER+ breast tumours (Silvestrini *et al*, 1994; Lipponen *et al*, 1995). This ‘paradoxical’ favourable prognostic effect of BCL2 in breast cancer could be related to its non-apoptotic functions (Pietenpol *et al*, 1994; O'Reilly *et al*, 1996). Increased expression of BCL2 protein may also disrupt the balance with other members of the BCL2 family, including the expression of pro-apoptotic proteins (Cory *et al*, 2003).

The exact mechanism of differential BCL2 protein expression in breast cancer is complex. BCL2 is expressed in normal breast glandular epithelium and is known to be upregulated by oestrogen, possibly as a direct result of transcriptional induction (Wang and Phang, 1995; Leung and Wang, 1999). We show that, in cancers, BCL2 positivity is not simply a surrogate for ER positivity: 14% of BCL2+ tumours were ER− and 31% of BCL2− tumours were ER+. BCL2 amplification/copy number gain is rare and correlation between transcript and protein levels in breast cancer is not linear (unpublished observations), suggesting post-transcriptional regulation. Therefore, although in Oncotype DX (Paik *et al*, 2004), BCL2 is one of the 21 genes in the prognostic signature, measurement of BCL2 protein expression may provide prognostic information that is not identical.

The prognostic value of BCL2 was present across molecular subtypes (ER+/luminal, HER2+, HER2− and triple negative), an important new observation, and was independent of tumour size, grade and stage. Women with ER+/BCL2− disease were found to have a worse prognosis than those with ER−/BCL2+ disease. The interaction between treatment and the prognostic role of BCL2 was also addressed, showing that the prognostic impact of BCL2 is independent of adjuvant therapy received. Finally, BCL2 is an important prognostic variable in a risk prediction setting and improves the performance of prediction algorithms such as AOL. Novel markers that could be used to save women from unnecessary cytotoxic adjuvant therapy are urgently needed and BCL2 provides valuable additional prognostic information to guide clinical decision making in this setting.

In summary, this large analysis establishes BCL2 as an independent and powerful prognostic protein marker in early-stage breast cancer. BCL2 provides prognostic information in all subgroups defined by other prognostic factors. Thus, it could be used with prediction algorithms such as AOL to improve the ability to discriminate risk groups, by simply using the HRs and the prevalence of BCL2 positivity in each subgroup we report. Indeed, the assignment of patients to risk groups is significantly altered by BCL2 expression (see Figures 2, 3, 4). The exact way to apply BCL2 testing for risk stratification and the approach to standardise BCL2 immunohistochemistry for this application will now require rigorous prospective assessment, but, given our findings reported here, this is feasible.

## Figures and Tables

**Table 1:** Characteristics of cases by study

	**SEARCH**	**NBCS**	**UBCBCS**	**MCCS**	**BCCA**	**Total**
No. of patients	3420	1926	976	850	4040	11 212
Mean age (years, range)	52 (23–69)	54 (18–70)	48 (22–90)	60 (41–79)	59 (23–95)	55 (18–95)
Mean follow-up (years, range)	7.3 (0.5–15.9)	5.2 (0–12.6)	9.7 (0–39.4)	7.2 (0–16.2)	10.9 (0–18.5)	8.4 (0–39.4)
Number of deaths (%)	469 (14)	416 (22)	492 (50)	133 (16)	1136 (28)	2646 (24)
Annual mortality (%)	2.3	4.2	5.8	1.7	2.7	3.0
						
*Tumour size (n* (%))						
<2 cm	1272 (57)	1023 (53)	114 (12)	543 (66)	1594 (40)	5133 (52)
2–4.9 cm	872 (39)	853 (44)	569 (62)	262 (32)	2108 (53)	4092 (42)
⩾5 cm	93 (4)	42 (2)	238 (26)	23 (3)	301 (8)	568 (6)
						
*Grade (n* (%))						
1	624 (22)	363 (19)	68 (9)	171 (22)	211 (5)	1436 (14)
2	1350 (47)	645 (34)	234 (32)	348 (44)	1578 (41)	4108 (41)
3	884 (31)	907 (47)	425 (59)	265 (34)	2067 (54)	4522 (45)
						
*Nodal status (n* (%))						
Negative	1346 (62)	1218 (64)	238 (30)	521 (66)	2156 (55)	5397 (57)
Positive	834 (38)	695 (36)	562 (70)	228 (30)	1741 (45)	4031 (43)
						
*ER status (n* (%))						
Negative	657 (20)	536 (30)	491 (55)	129 (28)	1224 (31)	3050 (29)
Positive	2681 (80)	1253 (70)	409 (45)	333 (72)	2785 (69)	7542 (71)
						
*PR status (n* (%))						
Negative	708 (30)	772 (44)	503 (60)	222 (48)	1758 (49)	3692 (44)
Positive	1621 (70)	993 (56)	338 (40)	237 (51)	1843 (51)	5110 (56)
						
*HER2 status (n* (%))						
Negative	1329 (89)	1341 (92)	637 (71)	407 (88)	3355 (87)	7006 (86)
Positive	172 (11)	116 (8)	256 (29)	54 (12)	506 (13)	1094 (14)
						
*BCL2 status (n* (%))						
Negative	488 (24)	270 (27)	258 (35)	198 (43)	971 (26)	2132 (27)
Positive	1509 (76)	714 (73)	471 (65)	265 (57)	2743 (74)	5671 (73)

Abbreviations: BCCA=British Columbia Cancer Agency Case Series; BCL2=B-cell lymphoma 2; ER=oestrogen receptor; HER2=human epidermal growth factor receptor 2; MCCS=Melbourne Collaborative Cohort Study; NBCS=Nottingham Breast Cancer Series; PR=progesterone receptor; SEARCH=Study of Epidemiology and Risk Factors in Cancer Heredity; UBCBCS=University of British Columbia Breast Cancer Series.

**Table 2:** Cox model incorporating time-dependent effects of prognostic variables

	**HR**	***B*_1_**	**95% CI**	***P*-value**	***T***	***B*_2_**	**95% CI**	***P*-value**
*Univariate analysis*								
Size (*n*=9793)	2.74	1.01	2.39–3.14	<0.001	0.79	−0.24	0.72–0.86	<0.001
Grade (*n*=10 066)	5.30	1.67	4.40–6.40	<0.001	0.53	−0.63	0.47–0.59	<0.001
Nodal status (*n*=9428)	4.02	1.39	3.30–4.90	<0.001	0.76	−0.27	0.67–0.87	<0.001
ER (*n*=10 592)	0.17	−1.77	0.14–0.20	<0.001	2.49	0.91	2.24–2.76	<0.001
PR (*n*=8802)	0.15	−1.90	0.13–0.19	<0.001	2.45	0.90	2.19–2.75	<0.001
HER2 (*n*=8100)	2.85	1.05	2.34–3.48	<0.001	0.70	−0.36	0.61–0.80	<0.001
BCL2 (*n*=7803)	0.21	−1.56	0.17–0.25	<0.001	2.02	0.70	1.78–2.31	<0.001
								
*Multivariate analysis: Model 1 (n*=*6738)*								
ER	0.39	−0.94	0.29–0.51	<0.001	1.99	0.69	1.65–2.41	<0.001
PR	0.30	−1.20	0.23–0.40	<0.001	1.76	0.57	1.49–2.08	<0.001
HER2	1.50	0.41	1.20–1.89	<0.001	0.97	−0.03	0.82–1.14	0.69
BCL2	0.62	−0.48	0.48–0.80	<0.001	1.09	0.09	0.92–1.29	0.32
								
*Multivariate analysis: Model 2 (n*=*5445)*								
Size	1.50	0.41	1.37–1.64	<0.001	NA			
Grade	2.89	1.06	2.18–3.80	<0.001	0.67	−0.40	0.57–0.79	<0.001
Nodal status	3.21	1.17	2.50–4.12	<0.001	0.80	−0.22	0.68–0.94	0.006
ER	0.41	−0.89	0.30–0.54	<0.001	2.07	0.73	1.70–2.50	<0.001
PR	0.33	−1.11	0.24–0.46	<0.001	1.69	0.52	1.41–2.03	<0.001
HER2	1.31	0.27	1.15–1.50	<0.001	NA			
BCL2	0.76	−0.25	0.66–0.88	<0.001	NA			

Abbreviations: *B*_1_=log(hazard ratio); *B*_2_=log(time effect); BCL2=B-cell lymphoma 2; CI=confidence interval; ER=oestrogen receptor; HER2=human epidermal growth factor receptor 2; HR=hazard ratio (mortality); NA=not applicable; PR=progesterone receptor; *T*=time effect.

*B*_2_ is positive when the HR increases with time and negative when the HR decreases with time.

Tumour size and grade were treated as continuous variables in the univariate and multivariate analyses.
