# BCL2 in breast cancer: a favourable prognostic marker across molecular subtypes and independent of adjuvant therapy received

**DOI:** 10.1038/sj.bjc.6605921

**Published:** 2010-09-28

**Authors:** S-J Dawson, N Makretsov, F M Blows, K E Driver, E Provenzano, J Le Quesne, L Baglietto, G Severi, G G Giles, C A McLean, G Callagy, A R Green, I Ellis, K Gelmon, G Turashvili, S Leung, S Aparicio, D Huntsman, C Caldas, P Pharoah

**Correction to**: *British Journal of Cancer* (2010) **103:** 668–675; doi:10.1038/sj.bjc.6605736

Upon publication of the above paper in Volume 103, Issue 5, the authors noticed an error in Table 1, wherein some of the totals in the final column were incorrect.

The correct Table 1 is as shown here.

## Figures and Tables

**Table 1:** 

	**SEARCH**	**NBCS**	**UBCBCS**	**MCCS**	**BCCA**	**Total**
No. of patients	3420	1926	976	850	4040	11 212
Mean age years (range)	52 (23–69)	54 (18–70)	48 (22–90)	60 (41–79)	59 (23–95)	55 (18–95)
Mean follow-up (years, range)	7.3 (0.5–15.9)	5.2 (0–12.6)	9.7 (0–39.4)	7.2 (0–16.2)	10.9 (0–18.5)	8.4 (0–39.4)
Number of deaths (%)	469 (14)	416 (22)	492 (50)	133 (16)	1136 (28)	2646 (24)
Annual mortality (%)	2.3	4.2	5.8	1.7	2.7	3.0
						
*Tumour size,* n (%)						
<2 cm	1272 (57)	1023 (53)	114 (12)	543 (66)	1594 (40)	4546 (46)
2–4.9 cm	872 (39)	853 (44)	569 (62)	262 (32)	2108 (53)	4664 (47)
⩾5 cm	93 (4)	42 (2)	238 (26)	23 (3)	301 (8)	697 (7)
						
*Grade,* n (%)						
1	624 (22)	363 (19)	68 (9)	171 (22)	211 (5)	1437 (14)
2	1350 (47)	645 (34)	234 (32)	348 (44)	1578 (41)	4155 (41)
3	884 (31)	907 (47)	425 (59)	265 (34)	2067 (54)	4548 (45)
						
*Nodal status,* n (%)						
Negative	1346 (62)	1218 (64)	238 (30)	521 (66)	2156 (55)	5479 (57)
Positive	834 (38)	695 (36)	562 (70)	228 (30)	1741 (45)	4060 (43)
						
*ER status,* n (%)						
Negative	657 (20)	536 (30)	491 (55)	129 (28)	1224 (31)	3037 (29)
Positive	2681 (80)	1253 (70)	409 (45)	333 (72)	2785 (69)	7461 (71)
						
*PR status,* n (%)						
Negative	708 (30)	772 (44)	503 (60)	222 (48)	1758 (49)	3963 (44)
Positive	1621 (70)	993 (56)	338 (40)	237 (51)	1843 (51)	5032 (56)
						
*HER2 status,* n (%)						
Negative	1329 (89)	1341 (92)	637 (71)	407 (88)	3355 (87)	7069 (86)
Positive	172 (11)	116 (8)	256 (29)	54 (12)	506 (13)	1104 (14)
						
*BCL2 status,* n (%)						
Negative	488 (24)	270 (27)	258 (35)	198 (43)	971 (26)	2185 (27)
Positive	1509 (76)	714 (73)	471 (65)	265 (57)	2743 (74)	5702 (73)

