# Supplementary figures and images for: BCL2 in breast cancer: a favourable prognostic marker across molecular subtypes and independent of adjuvant therapy received
Source: Br J Cancer. 2010 Jul 27;103(5):668–75. doi: 10.1038/sj.bjc.6605736 (PMC2938244; doi:10.1038/sj.bjc.6605736)

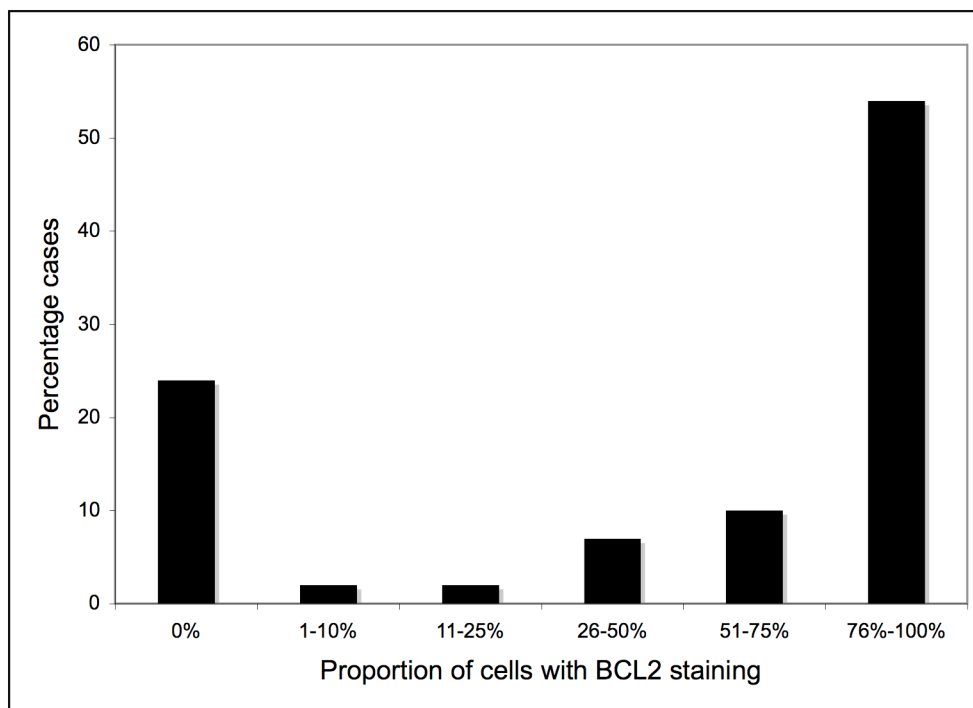

**Supplementary Figure 1**

Supplement: Supplementary Figure 1 [file 6605736x1.pdf]

**A**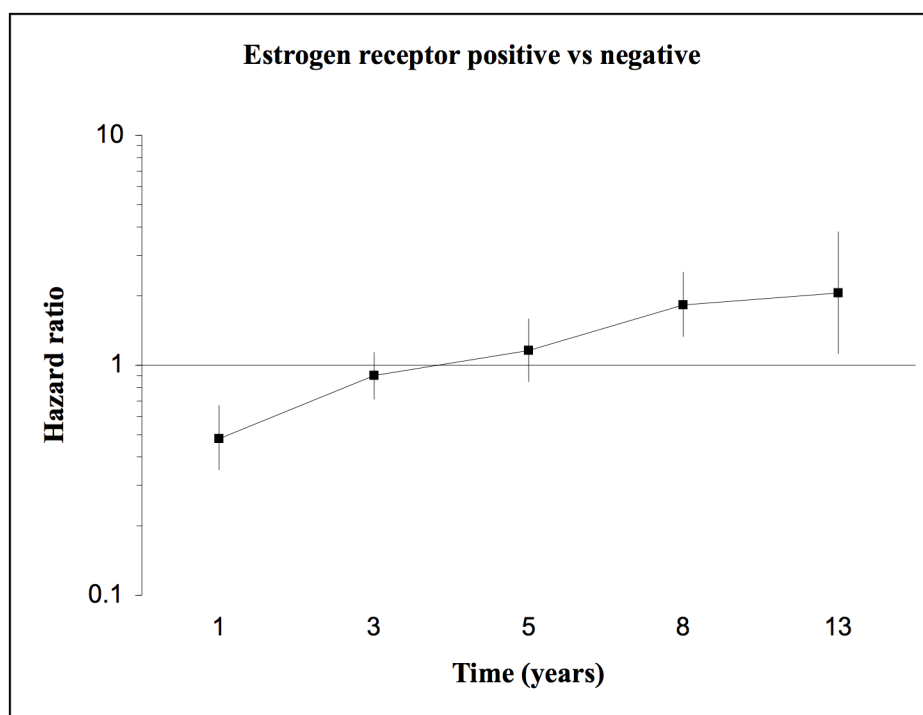**B**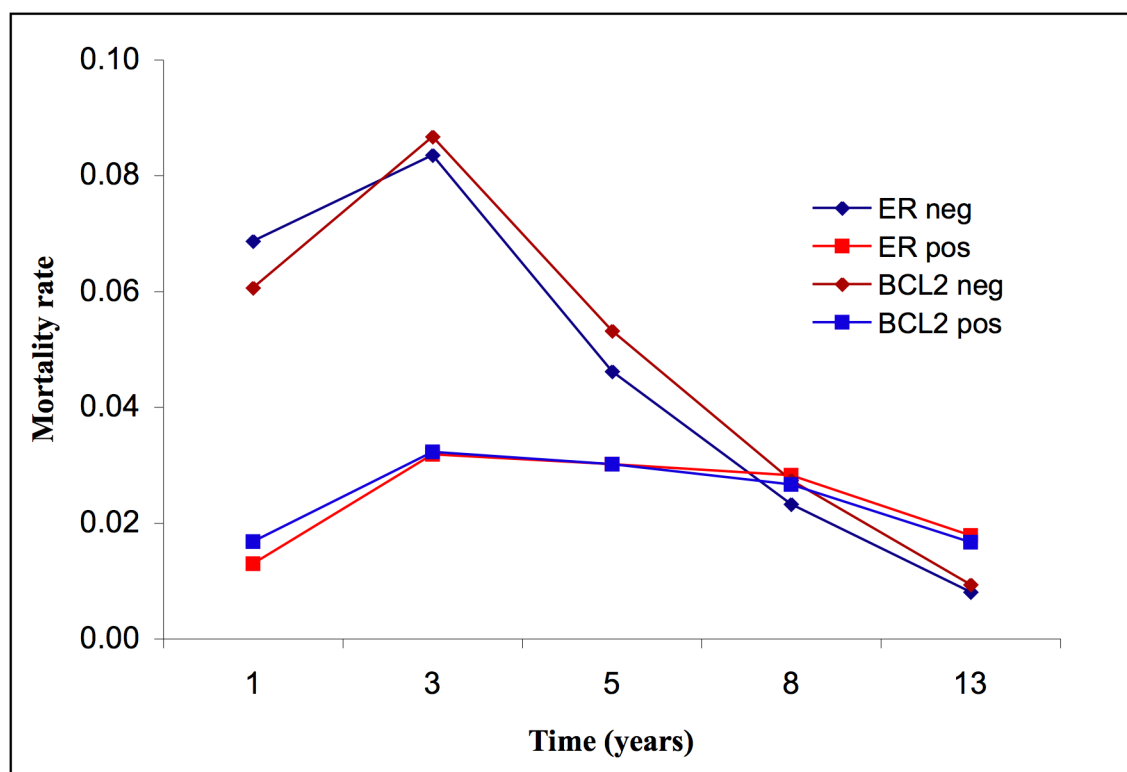

Supplementary Figure 2

Supplement: Supplementary Figure 2 [file 6605736x2.pdf]
