# Supplementary material for: BCL2 in breast cancer: a favourable prognostic marker across molecular subtypes and independent of adjuvant therapy received
Source: Br J Cancer. 2010 Jul 27;103(5):668–75. doi: 10.1038/sj.bjc.6605736 (PMC2938244; doi:10.1038/sj.bjc.6605736)
Supplement: Supplementary Methods [file 6605736x3.doc]

**SUPPLEMENTARY INFORMATION**

**SUPPLEMENTARY METHODS**

**Immunostaining**

Immunostaining was performed using the following antibodies: ER (Novocastra clone

6F11 for SEARCH, NBCS and UBCBCS; LabVision clone SP1 for BCCA and MCCS),

PR (Dako clone PgR 636 for SEARCH, NBCS, UBCBCS and MCCS; Ventana clone

1E2 for BCCA), HER2 (Dako clone A0485 for SEARCH and MCCS; Novocastra clone

CE-356 for NBCS and UBCBCS; LabVision clone SP3 for BCCA) and BCL2 (Dako

clone 124 for all studies). All antibodies were initially optimized on control tissue and

positive and negative controls were processed in parallel to the tissue microarray or

whole tissue sections. Tissue sections were cut at 3-4μm for immunostaining.

For the SEARCH series, immunostaining for ER, PR and BCL2 was performed using the BOND-maXTM automated immunostainer (Leica Microsystems) using the following antibody concentrations: ER (1:70), PR (1:50) and BCL2 (1:200). HER2 staining was performed using the HercepTest^TM^ kit on the Dako autostainer (Dako). For the NBCS and UBCBCS series, immunostaining was performed using the TechMate automated immunostainer (Dako, Ely, Cambridgeshire, United Kingdom) using the following antibody concentrations: ER (1:30), PR (1:50), BCL2 (1:20) and HER2 (1:40) (Calla*gy et* al, 2006). For the MCCS series, immunostaining was performed using the Lab Vision Autostainer 360-2D (Lab Vision, California) using the following antibody concentrations: ER (1/250), PR (1/100) and BCL2 (1:800), HER2 (1:1000). For the BCCA series, immunostaining for ER, PR and HER2 was performed using the Ventana Benchmark XT staining system (Ventana Medical Systems, Tucson, AZ, USA) using the following antibody concentrations: ER (1:250), PR (prediluted by supplier, Ventana) and HER2 (1;100). Immunostaining for BCL2 in the BCCA series was performed as described for the SEARCH series.

**Scoring**

*SEARCH and MCCS*

For BCL2, the intensity (0=no staining, 1=weak staining, 2=moderate staining, 3=strong

staining) and the percentage of cells with positive cytoplasmic staining were recorded.

All cases with staining in >10 percent of cells were considered positive. This cut-off was

established in the previous analysis of the NBCS and UBCBCS series (Calla*gy et* al, 2006). ER and PR were scored using the Allred scoring system and all cases with a score of >2 were considered positive. HER2 was scored according to HercepTest criteria as follows: 0=no staining or faint incomplete staining in <10% cells, 1+=faint incomplete staining in >10% cells, 2+= weak to moderate complete staining in >10% cells, 3+= strong complete staining in >10% cells. All cases with a score of 2+ or 3+ were considered positive. Scoring of the SEARCH series was performed by S.J.D. and scoring of the MCCS was performed by C.M.

*BCCA*

BCL2 scoring was performed as described for the SEARCH series. In a total of 500 cases

from this series BCL2 scoring was independently performed by S.J.D. in Cambridge and

G.T. in Vancouver (kappa 0.9 for dichotomous score). Scoring of the whole BCCA

series was then performed by G.T. Immunohistochemical status of ER and PR was determined by the percentage of tumor cell nuclear positivity, and scored as negative (< 1%); positive 1+ (1% to 25%); positive 2+ (> 25% to 75%); or positive 3+ (> 75%). For all analyses, scores for ER and PR were dichotomized at >1% = ER or PR positive. HER2 status was determined by both immunohistochemistry and fluorescence in situ hybridisation (FISH). Tumors were positive if scored as 3+ based on HercepTest criteria, or amplification ratio >2.0 by FISH when the immunohistochemistry score was 2+.

*NBCS and UBCBCS*

For BCL2, both the intensity of staining (0-4) and the percentage of positive cells were

recorded. All cases with staining in >10 percent of cells were considered positive (Calla*gy et* al, 2006). In our analysis, in order to examine prognostic significance according to BCL2 staining intensity, the original NBCS and UBCBCS intensity scores 3 and 4 were combined into a single score to represent strong staining. This allowed direct comparison with the BCL2 intensity scores in the SEARCH, BCCA and MCCS series. ER and PR in the NBCS and UBCBCS series were scored using a semiquantitative method as follows: 0=no staining; 1=<10%; 2=11-25%; 3=26-50%; 4=51-75%; 5=>75%. All cases with staining in >10 percent of cells were considered positive. HER2 was scored according to the HercepTest criteria with a score of 3+ considered positive. Scoring of the NBCS and UBCBCS series was performed by G.C.

**Statistical Analysis**

The statistical analysis was conducted in concordance with recently published guidelines for the development of prognostic models in breast cancer (Altman, 2009). Cox regression analysis stratified by study was performed to determine the effect of each prognostic factor and marker on mortality after diagnosis. Time under observation began on the date of diagnosis for cases from NBCS, UBCBCS, BCCA and MCCS and on date of entry into the study for SEARCH. The end-point of interest was death from breast cancer which was available for SEARCH, NBCS and BCCA. Death from any cause was used as the end-point as a proxy for breast cancer specific mortality for UBCBCS and MCCS, as cause-specific mortality data were not available for these studies. Follow-up was censored on the date of death for cases from SEARCH, NBCS and BCCA that died from causes other than breast cancer, or, if death did not occur, on date last known alive or at fifteen years after diagnosis, whichever came first. Date of study entry was used to determine time under observation rather than date of diagnosis for the SEARCH study to allow for the fact that SEARCH is an ongoing epidemiology study and some cases are recruited after diagnosis – left truncation. This provides an unbiased estimate of the hazard ratio if the Cox model is correctly specified (Azza*to et* al, 2009). This is not relevant for the other studies in which cases were ascertained at the time of diagnosis.

Node status and immunohistochemical markers were all treated as dichotomous variables

(positive vs. negative), grade was categorised into three groups (well differentiated (grade

1), moderately differentiated (grade 2) and poorly differentiated (grade 3)) and tumor size

was similarly categorised into three groups (<2cm, 2-4.9cm and ≥5cm). The ordinal

variables were treated as continuous variables in the regression models.

Using the Cox model allowing for time-dependence, the HR was calculated over the 15

year follow up period. Confidence intervals were estimated by simulation. Each

parameter was estimated 1000 times using coefficient values drawn from distributions

based on the point estimate of each regression coefficient and standard error, accounting

for the covariance of the estimates. The 95 percent confidence interval was then derived

from the 2.4 and 97.5 centiles of the simulated parameter distribution.

The performance of BCL2 as a prognostic marker for risk prediction was assessed by examining its validity in predicting the expected number of deaths in the series. A receiver operating characteristic (ROC) curve was used to explore the performance of the prognostic model with and without the inclusion of BCL2 (Pe*pe et* al, 2004). A comparison of the performance of the risk prediction algorithm Adjuvant! Online with and without the inclusion of BCL2 was also performed. In order to do this Adjuvant! Online requires that the hazard ratio for the high risk group and the prevalence of the high risk group are added as parameters to the model. We used a prevalence of 27% for BCL2 negativity and a hazard ratio of 1.32 (0.76^-1^). A relative utility curve was generated to compare the performance of Adjuvant! Online with and without the inclusion of BCL2 (Baker, 2009). The relative utility is the fraction of the expected utility of perfect prediction achieved at the optimal cut point for a risk prediction model.

All analyses were performed using Intercooled Stata, version 9 (Stata Corp).

**Supplementary Methods References**

Altman DG (2009) Prognostic models: a methodological framework and review of models for breast cancer. *Cancer Invest* **27:** 235-43

Azzato EM, Greenberg D, Shah M, Blows F, Driver KE, Caporaso NE, Pharoah PD (2009) Prevalent cases in observational studies of cancer survival: do they bias hazard ratio estimates? *Br J Cancer* **100:** 1806-11

Baker SG (2009) Putting risk prediction in perspective: relative utility curves. *J Natl Cancer Inst* **101:** 1538-42

Callagy GM, Pharoah PD, Pinder SE, Hsu FD, Nielsen TO, Ragaz J, Ellis IO, Huntsman D, Caldas C (2006) Bcl-2 is a prognostic marker in breast cancer independently of the Nottingham Prognostic Index. *Clin Cancer Res* **12:** 2468-75

Pepe MS, Janes H, Longton G, Leisenring W, Newcomb P (2004) Limitations of the odds ratio in gauging the performance of a diagnostic, prognostic, or screening marker. *Am J Epidemiol* **159:** 882-90

**SUPPLEMENTARY TABLES**

**Supplementary Table 1: Compliance of analysis with REMARK criteria**

**INTRODUCTION**

**1.** The marker examined, BCL2, and the study objectives are defined in the introduction.

**MATERIALS AND METHODS**

**Patients**

**2.** Characteristics of the study patients are summarised in Table 1. Details of the

individual studies to which the patients were recruited are detailed in Supplementary

table 1. The inclusion criteria are specified in the materials and methods.

**3.** Treatment information was available on a proportion of study participants (adjuvant

chemotherapy n=6,128; adjuvant endocrine therapy n=5,700).

**Specimen characteristics**

**4.** Formalin fixed paraffin embedded tissue which had been stored at individual

participating centres was used in the analysis. The preparation of the biological material

used in the analysis is described in the materials and methods (including supplementary

methods).

**Assay methods**

**5.**  The antibodies, immunostaining and scoring techniques used are described in the

materials and methods (including supplementary methods).

**Study design**

**6.** Cases were selected prospectively for each of the studies and biological material was

collected in parallel for the purpose of biomarker assessment. The time periods from

when the cases were taken are specified in Supplementary table 1. The follow up period

for all cases is summarised in Table 1.

**7.** The only clinical endpoint examined was survival as specified in the materials and

methods (including supplementary methods).

**8.** All candidate variables included in the models (ER, PR, HER2, BCL2, tumor grade,

tumor size and lymph node status) were outlined in the materials and methods.

**9.** The large sample size of over 11,000 women was chosen to provide adequate

statistical power to answer the study objectives.

**Statistical analysis methods**

**10.** The statistical methods (including supplementary methods) detail all model-building

issues and how model assumptions were verified.

Information on pathological characteristics were not available for some participants.

Similarly, ER, PR, HER2 and BCL2 status was not assessable for all participants either

because staining was not performed, missing cores were present on the tumor

microarrays or the selected cores contained no invasive tumor on tissue micorarrays. The

proportion of data missing for each of the variables examined was as follows: tumor size

n=1,419, grade n=1,146, nodal status n=1,784, ER status n=620, PR status n=2,410,

HER2 status n=3,112 and BCL2 status n=3,409. Despite the missing information,

sufficient data was available to allow the univariate, multivariate and subgroup analyses

to be performed with adequate statistical power, given the overall large size of the series.

**11.** Cutpoint determination for defining marker positivity was outlined in the materials

and methods (provided in supplementary methods).

**RESULTS**

**Data**

**12.** The number of patients included in each stage of the analysis is detailed in Table 2.

**13.** Basic demographic characteristics of study participants are summarised in Table 1.

**Analysis and presentation**

**14.** The relationship of the marker, BCL2, to standard prognostic variables is detailed in

the results.

**15.** Univariate analysis showing the relationship between BCL2 and outcome with the

estimated effect (i.e. hazard ratio) is shown in the results (Table 2). The univariate

analysis is also presented for all other variables analysed. Kaplan-Meier plots are shown

in Figures 1 and 3.

**16.** Multivariate analysis reporting the estimated effect (i.e. hazard ratio) with confidence

intervals for BCL2 and all other variables in the models are included in Table 2.

**17.** The estimated effect (i.e. hazard ratio) and confidence intervals from a multivariate

analysis including BCL2 and standard prognostic variables is included in Table 2.

**18.** Further investigations were not relevant to the current analysis.

**DISCUSSION**

**19.** The results were interpreted in the context of the study objectives and other relevant

studies in the discussion. The study limitations were also outlined.

**20.** Implications for future research and clinical value were highlighted in the discussion.

**Supplementary Table 2: Description of the Study Populations**

| **Study** | **Description** | **No. of breast cancer cases with tumor tissue available** |
| --- | --- | --- |
| **SEARCH**  Study of Epidemiology and Risk Factors in Cancer Hereditary  **NBCS**  Nottingham Breast Cancer Series  **UBCBCS**  University of British Columbia Breast Cancer Series (Canada)  **MCCS**  Melbourne Collaborative Cohort Study  **BCCA**  British Columbia Cancer Agency Case Series | England population based series (case control study).  2 groups of cases identified through the Eastern Cancer Registration and Information Centre; 1) prevalent cases diagnosed age <55 from 1991-1996 and alive when study started in 1996; 2) incident cases diagnosed age <70 after 1996.  England series of primary operable breast carcinoma patients presenting from 1986 to 1998 and entered into the Nottingham Tenovus Primary Breast Carcinoma Series.  Canadian case series of women with stage I to III breast cancer who participated in four different British Columbia Cancer Agency clinical trials between 1970 and 1990 and all received chemotherapy.  Australian population based prospective cohort study. Subjects were recruited between 1990 and 1994 via the electoral roll and community advertisements.  Canadian case series of women diagnosed with invasive breast cancer between 1986 to 1992 and identified through the British Columbia Cancer Agency | 3,420  1,926  976  850  4,040 |

**Supplementary Table 3: Performance of the prognostic model across four studies (SEARCH, NBCS, MCCS and BCCA)**

|  | **HR^*^** | **B1^**^** | **95% CI** | **P-value** | **T^†^** | **B2^††^** | **95% CI** | **P-value** |
| --- | --- | --- | --- | --- | --- | --- | --- | --- |
| **Multivariate Analysis** | | | | | | | | |
| **Size** | 1.52 | 0.42 | 1.38-1.67 | <0.001 | NA |  |  |  |
| **Grade** | 2.89 | 1.06 | 2.13-3.94 | <0.001 | 0.67 | -0.40 | 0.56-0.80 | <0.001 |
| **Nodal status** | 3.29 | 1.19 | 2.54-4.30 | <0.001 | 0.80 | -0.22 | 0.68-0.95 | 0.009 |
| **ER** | 0.38 | -0.97 | 0.28-0.52 | <0.001 | 2.20 | 0.79 | 1.80-2.73 | <0.001 |
| **PR** | 0.31 | -1.16 | 0.22-0.44 | <0.001 | 1.68 | 0.55 | 1.42-2.10 | <0.001 |
| **HER2** | 1.27 | 0.27 | 1.13-1.51 | <0.001 | NA |  |  |  |
| **BCL2** | 0.79 | -0.23 | 0.69-0.93 | 0.003 | NA |  |  |  |

**^*^**HR = hazard ratio (mortality); ^**^B_1_= log (hazard ratio); ^†^T = time effect; **^††^**B_2_ = log (time effect).

B_2_ is positive when the HR increases with time and negative when the HR decreases with time.

NA = not applicable

**SUPPLEMENTARY FIGURES**

**Supplementary Figure 1: Distribution of percentage positivity of BCL2 staining**

The distribution of the percentage of cells with positive BCL2 staining is shown for the whole series

**Supplementary Figure 2: Time dependence of prognostic factors ER and BCL2**

A. Hazard ratio over time for ER.

Hazard ratios were calculated over the 15 year follow up period using the Cox model

allowing for time-dependence in multivariate analysis (Model 1, Table 2).

B. Annual mortality rate according to ER and BCL2 status.
